# Evaluating the Hemodynamical Response of a Cardiovascular System under Support of a Continuous Flow Left Ventricular Assist Device via Numerical Modeling and Simulations

**DOI:** 10.1155/2013/986430

**Published:** 2013-12-02

**Authors:** Selim Bozkurt, Koray K. Safak

**Affiliations:** Department of Mechanical Engineering, Yeditepe University, 34755 Kadikoy, Istanbul, Turkey

## Abstract

Dilated cardiomyopathy is the most common type of the heart failure which can be characterized by impaired ventricular contractility. Mechanical circulatory support devices were introduced into practice for the heart failure patients to bridge the time between the decision to transplant and the actual transplantation which is not sufficient due to the state of donor organ supply. In this study, the hemodynamic response of a cardiovascular system that includes a dilated cardiomyopathic heart under support of a newly developed continuous flow left ventricular assist device—Heart Turcica Axial—was evaluated employing computer simulations. For the evaluation, a numerical model which describes the pressure-flow rate relations of Heart Turcica Axial, a cardiovascular system model describing the healthy and pathological hemodynamics, and a baroreflex model regulating the heart rate were used. Heart Turcica Axial was operated between 8000 rpm and 11000 rpm speeds with 1000 rpm increments for assessing the pump performance and response of the cardiovascular system. The results also give an insight about the range of the possible operating speeds of Heart Turcica Axial in a clinical application. Based on the findings, operating speed of Heart Turcica Axial should be between 10000 rpm and 11000 rpm.

## 1. Introduction

Dilated cardiomyopathy (DCM) is the most common type of nonischemic cardiomyopathy and a major cause of congestive heart failure [1, 2]. It leads to the weakening and enlargement of the heart and impairs its function to pump blood. It can be caused by genetic, viral, immune, alcoholic-toxic, or unknown factors. It can be associated with a different cardiovascular disease. Symptoms of this disease are fatigue, weakness, and systemic or pulmonary emboli [3]. In a DCM patient, reduced contractile function causes a dilatation in the ventricle cavity volume and a thinning in the wall volume. Left ventricular systolic pressure decreases while diastolic pressure increases. With the reduced contractility and systolic pressure, cardiac output decreases. To maintain the perfusion, the arteries and the veins constrict and the blood volume increases. With progression of the disease, systemic arterial pressure decreases and this leads to an increased workload on the right ventricle. Right ventricular systolic pressure and pulmonary arterial pressure increase accordingly, resulting in pulmonary hypertension [4–13].

DCM is conventionally treated with inotropic support, diuretics, and moderate exercise. When these methods fail, especially towards the final stage of the disease, heart transplantation is called for. With the current state of donor organ supply, many patients would not be treated, due to lack of a fitting donor organ. Furthermore, availability of a donor heart for urgent patients is not always timely. For these patients, to bridge the time between the decision to transplant and the actual transplantation, cardiac assist devices have been introduced into clinical practice [3].

Ventricular assist devices (VADs) are the devices supporting the failing ventricle partially or completely. Because of the native heart being kept in place in a VAD patient, the VADs can be used for bridge to transplantation or as a destination therapy. In some cases, the heart may regain its original function, so the pump support is only temporary, until the failing heart has recovered [14]. Generally, the VADs are classified as intra-aortic balloon pumps, continuous flow left ventricular assist devices (CF-LVADs) and the pulsatile ventricular assist devices (P-VADs). The VAD therapy is suggested when cardiac index is less than 2 L/min/m^2^, systolic arterial pressure is less than 90 mmHg, atrial pressure is higher than 20 mmHg, or the systemic vascular resistance is higher than 1.57 mmHgs/mL [15, 16]. CF-LVADs represent the new generation of the mechanical assist device treatment of the heart failure. There are a number of advantages of these devices over P-VADs such as having smaller size, less moving parts, and smaller blood contact surface. Overall, the good durability of the devices has led to acceptation of VAD support as a viable alternative for total heart transplantation.

In this study, we aim to evaluate the hemodynamical response of a DCM cardiovascular system model under support of a newly developed CF-LVAD-Heart Turcica Axial (HTA) [17]. The results also give an insight about the possible operating speed of this CF-LVAD in a patient.

## 2. Materials and Methods

The baseline geometry of HTA includes an inducer at the inlet, a diffuser at the outlet, and a rotating impeller. These components were expressed using eleven design parameters. The design parameters were optimized using genetic algorithms in order to increase pressure rise at the outlet of the pump and reduce the wall shear stresses on the surface of the components for 6 L/min flow rate through the pump. The optimized geometries were assessed employing Computational Fluid Dynamics (CFD) analyses. The geometry generates the highest pressure rise at the outlet of the pump and lower wall shear stress on the components was selected as HTA CF-LVAD. The detailed information about the shape optimization and CFD analyses can be found in [17, 22].

A model was chosen to estimate the pressure head of HTA using pump flow rate and operating speed and the coefficients were estimated using the results of the CFD analyses to describe HTA. This model is given as follows:

(1) $$\begin{array}{c} \Delta p_{\text{HTA}}=k_{1}Q_{\text{HTA}}+k_{2}n_{\text{HTA}}^{2}. \end{array}$$

**Figure 1:**
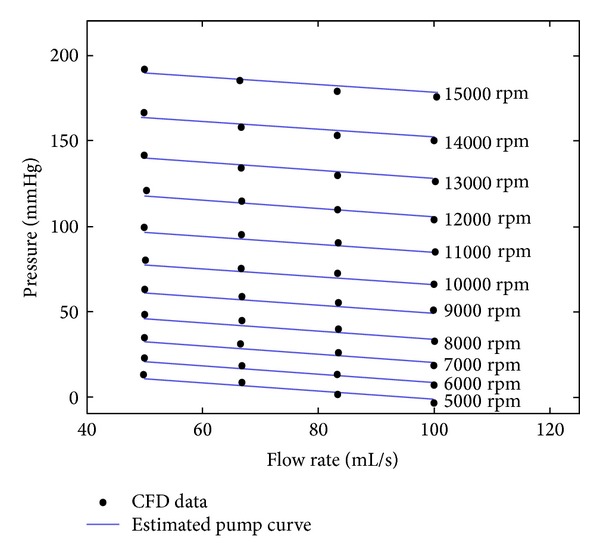
Data from the CFD analyses and the estimated HTA flow rate-pressure curves using (1).

Here,  Δ*p*_HTA_  is the pressure difference across HTA and  *Q*_HTA_  and  *n*_HTA_  are the flow rate through HTA and the operating speed of HTA, respectively.  *k*_1_  and  *k*_2_  are the estimated coefficients. The least squares approximation was used to estimate  *k*_1_  and  *k*_2_. Estimated values of  *k*_1_  and  *k*_2_  were 0.2380 mmHg mL^−1^ and 0.0032 mmHg s^−2^ with a root mean square error of 1.41 mmHg. The detailed information about the parameter estimation is given in [23]. Data from the CFD analyses and the estimated pump performance curves are given in Figure 1.

The cardiovascular system model includes ventricles, atria, heart valves, systemic arteries, systemic veins, pulmonary arteries and pulmonary veins. The ventricles were modeled using a time varying elastance (*E*) model [18]. This model is described by an activation function (*e*) and characteristic ventricular elastance of end-systole (*E*_*s*_) and end-diastole (*E*_*d*_). One has the following:

(2) $$\begin{array}{c} E=E_{d}\;+\frac{\left(E_{s}-E_{d}\right)e\left(t\right)}{2},\\ e\left(t\right)=\{\begin{array}{cc} 1-\cos\left(\left(\frac{t}{T_{1}}\right)\pi \right), & t<T_{1},\\ 1+\cos\left(\left(\frac{\left(t-T_{1}\right)}{\left(T_{1}-T_{2}\right)}\right)\pi \right), & T_{1}\leq t<T_{2},\\ 0, & T_{2}\leq t<T. \end{array} \end{array}$$

Here,  *t*  is the instantaneous time,  *T*_1_  and  *T*_2_  are the time instants, when the activation function becomes maximum and minimum, and  *T*  is the duration of a heartbeat. Left and right ventricles were modeled using the same equation with different  *E*_*s*_  values [18]. Pressure over a cardiac cycle in the left ventricle was modeled using the left ventricular volume (*V*_lv_), zero pressure filling volume of the left ventricle (*V*_0,lv_), and the elastance function (*E*_lv_). Model for the left ventricular pressure is given in

(3) $$\begin{array}{c} p_{\text{lv}}=\left(V_{\text{lv}}-V_{0,\text{lv}}\right)E_{\text{lv}}. \end{array}$$

A similar model was used for describing the right ventricular pressure considering the right ventricular elastance function, cavity volume, and zero pressure filling volume. Left ventricular volume (*V*_lv_) was described as the difference between flow rates through the mitral valve (*Q*_mv_) and aortic valve (*Q*_av_) as follows:

(4) $$\begin{array}{c} V_{\text{lv}}=Q_{\text{mv}}-Q_{\text{av}}. \end{array}$$

The blood vessels and the atria were modeled considering the analogy between the electrical systems and the circulatory system [24]. Model of the blood vessels includes the resistance, compliance and inertance properties. Atria were modeled as passive compliant chambers. Change of the pressure (*dp*_as_/*dt*) and flow rate (*dQ*_as_/*dt*) in the systemic arteries are given below.

(5) $$\begin{array}{c} \frac{dp_{\text{as}}}{dt}=\frac{\left(Q_{\text{av}}-Q_{\text{as}}\right)}{C_{\text{as}}},\\ \frac{dQ_{\text{as}}}{dt}=\frac{\left(p_{\text{as}}-p_{\text{vs}}-R_{\text{as}}Q_{\text{as}}\right)}{L_{\text{as}}}, \end{array}$$

where *Q*_av_  and  *Q*_as_  denote the flow rate through the aortic valve and the systemic arteries,  *p*_as_  and  *p*_vs_  denote the pressure in the systemic arteries and the systemic veins, and  *C*_as_,  *R*_as_, and  *L*_as_  are compliance, resistance and inertance in the systemic arterial model. Pressures and flow rates in the other compartments were modeled in a similar way. The heart valves were modeled as ideal diodes which allow only one-way blood flow. Flow rate through the aortic valve (*Q*_av_) is given in

(6) $$\begin{array}{c} Q_{\text{av}}=\{\begin{array}{cc} \frac{\left(p_{\text{lv}}\;-p_{\text{as}}\right)}{R_{\text{av}}}, & p_{\text{lv}}>p_{\text{as}},\\ 0, & p_{\text{lv}}\leq p_{\text{as}}. \end{array} \end{array}$$

**Figure 2:**
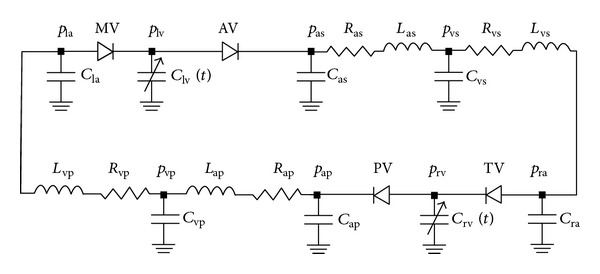
The electric analogue of the cardiovascular system.  *R*,  *L*  and  *C*  denote resistance, inertance, and compliance,  *p*  and  *Q*  denote pressure and flow rate, MV, AV, TV, and PV are the mitral, aortic, tricuspid and pulmonary valves, subscripts la, lv, ra, and rv represent the left atrium and ventricle and the right atrium and ventricle, as, vs, ap, and vp are the systemic arteries, the systemic veins, the pulmonary arteries, and the pulmonary veins, and ref and  *m*  represent reference and model.

Here,  *R*_av_  is the characteristic resistance of the aortic valve. Flow rate through the mitral, tricuspid, and pulmonary valves was described in a similar way. The electric analogue diagram of the cardiovascular system model is given in Figure 2 and the parameter values of resistance (*R*), compliance (*C*), inertance (*L*), and elastances (*E*) are presented in Table 1.

Dilated cardiomyopathy (DCM) was considered as the pathological case in the simulations. As mentioned before, in a DCM patient, contractions of the heart muscle weaken and left ventricular volume increases significantly while the ventricular stroke volume, systolic left ventricular and aortic pressures, cardiac output, and ejection fraction reduce [3]. Left ventricular systolic elastance was reduced to 0.5 mmHg mL^−1^ from 2.5 mmHg mL^−1^ in order to simulate a DCM condition in the cardiovascular system model.

HTA model and the circulatory system model were combined in order to simulate the CF-LVAD assistance by modifying the equations describing the left ventricular volume and systemic arterial pressure change as follows:

(7) $$\begin{array}{c} V_{\text{lv}}=Q_{\text{mv}}-Q_{\text{av}}-Q_{\text{HTA}},\\ \frac{dp_{\text{as}}}{dt}=\frac{\left(Q_{\text{av}}+Q_{\text{HTA}}\;-Q_{\text{as}}\right)}{C_{\text{as}}}. \end{array}$$

The term  Δ*p*_HTA_  in (1) becomes the pressure difference between the aorta and left ventricle because, the inlet of a CF-LVAD is attached to the apex of the left ventricle and the outlet of a CF-LVAD is attached to the aorta. So, (1) becomes

(8) $$\begin{array}{c} p_{\text{as}}-p_{\text{lv}}=k_{1}Q_{\text{HTA}}+k_{2}n_{\text{HTA}}^{2}. \end{array}$$

A baroreflex model was integrated into the model to regulate heart rate. Heart rate is regulated by complex interactions of sympathetic and vagal activities. Sympathetic activity causes a constriction in the peripheral vessels, a reduction in the abdominal blood flow, and an increase in the blood flow in the skeletal muscles. Vagal activity reduces the heart rate and has negative inotropic effects. In a healthy cardiovascular system resting heart rate is controlled by vagal mechanisms. In a failing heart, the vagal mechanism is less effective on the heart rate regulation [25]. The baroreflex model regulating the heart rate was taken from [26]. In this model, the afferent baroreflex pathway was defined using a sigmoidal function and linear first order dynamics as follows:

(9) $$\begin{array}{c} \tau_{p}\frac{dP}{dt}=p_{\text{as}}+\tau_{z}\frac{dp_{\text{as}}}{dt}-P, \end{array}$$

(10) $$\begin{array}{c} f_{cs}=\left(\frac{f_{\min}+f_{\max}e^{\left(P-p_{n}\right)/k_{a}}}{1+e^{\left(P-p_{n}\right)/k_{a}}}\right). \end{array}$$

Here,  *τ*_*p*_ and  *τ*_*z*_  are the time constants and  *P*  is the output of the linear system given in (9).  *f*_*cs*_  is the frequency of the spikes in the afferent fibers and  *f*_min⁡_  and  *f*_max⁡_  denote the minimum and maximum levels of the saturation function. Efferent sympathetic pathway was defined as an exponential function. To define the efferent vagal pathway a sigmoidal function was used. The model for the regulation effectors for the duration of a heartbeat is given as follows:

(11) $$\begin{array}{c} \sigma_{T,s}\left(t\right)=\{\begin{array}{cc} G_{T,s}\cdot \ln(f_{\text{es}}\left(t-D_{T,s}\right)\; & \\ -f_{\text{es},\min}+1), & f_{\text{es}}\leq f_{\text{es},\min},\\ 0, & f_{\text{es}}>f_{\text{es},\min}, \end{array}\\ \frac{d\Delta T_{s}\left(t\right)}{dt}=\frac{\left(-\Delta T_{s}\left(t\right)+\sigma_{T,s}\left(t\right)\right)}{\tau_{T,s}},\\ T=\Delta T_{s}+\Delta T_{v}+T_{0}. \end{array}$$

Here,  *σ*_*T*,*s*_  is the sympathetic activity, with strength  *G*_*T*,*s*_, time delay  *D*_*t*,*s*_, and time constant  *τ*_*T*,*s*_.  *f*_es_  denotes the sympathetic efferent pathway frequency and  *t*  is the instantaneous time. The vagal activity was described using a similar model with different parameter values [26].  Δ*T*_*s*_  and  Δ*T*_*v*_  denote sympathetic and vagal stimulation and  *T*_0_  denotes the heart period in absence of cardiac innervations, respectively.

## 3. Results and Discussion

The simulations were performed using Matlab Simulink R2010a. The set of the equations was solved using the ode15s solver. The maximum step size was 0.0005 s. The relative error tolerance was set to 0.001. All the simulations reached a periodic solution at a maximum of 30 s of simulation time. Results are given as 3 s time interval of the steady state periodic solution. Body surface area was assumed to be 1.8 m^2^ for calculating the cardiac index [27].

**Figure 3:**
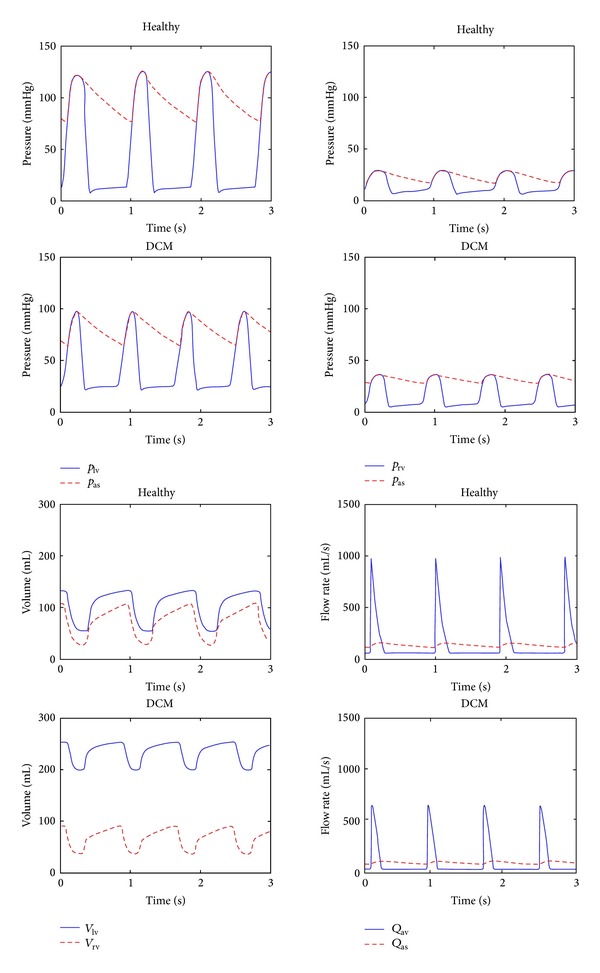
Pressures in the left ventricle (*p*_lv_), the systemic arteries (*p*_as_), the right ventricle (*p*_rv_), and the pulmonary arteries (*p*_ap_), volumes in the left ventricle (*V*_lv_) and the right ventricle (*V*_rv_), and flows through the aortic valve (*Q*_av_) and the systemic arteries (*Q*_as_) for the healthy and DCM models.

Simulations for the healthy and DCM cardiovascular system models were done to compare hemodynamics with the HTA assisted circulation. Ventricular pressures and volumes, systemic arterial pressures, and pulmonary arterial pressures are given in Figure 3.

Left ventricular peak pressure was 125 mmHg while the systemic arterial pressure varied between 78 mmHg and 125 mmHg over a cardiac cycle for the healthy model. The mean arterial pressure was 102 mmHg. Right ventricular peak pressure was 29 mmHg and the pulmonary arterial pressure was changing between 17 mmHg and 29 mmHg. Ventricular stroke volume was 78 mL over a cardiac cycle. Heart rate was 65 bpm and the cardiac output was 85 mL/s (5.10 L/min) in the simulations for the healthy model. In the DCM model, the peak left ventricular pressure reduced to 97 mmHg while there was an increase in the diastolic pressure. Systolic and diastolic arterial pressures decreased as well in the DCM model. Right ventricular peak pressure and pulmonary arterial systolic and diastolic pressures increased. Ventricular stroke volumes decreased with the lowered elastance in the DCM model while left ventricular volume was higher than 200 mL over a cardiac cycle. Heart rate was 75 bpm and the cardiac output was 66.17 mL/s (3.97 L/min). The amplitude of the aortic valve flow was around 1000 mL/s in the healthy model while it reduced to 650 mL/s in the DCM model. The variation of the systemic arterial flow was between 60 mL/s and 110 mL/s in the healthy model. In the DCM model it changed between 50 mL/s and 85 mL/s.

**Figure 4:**
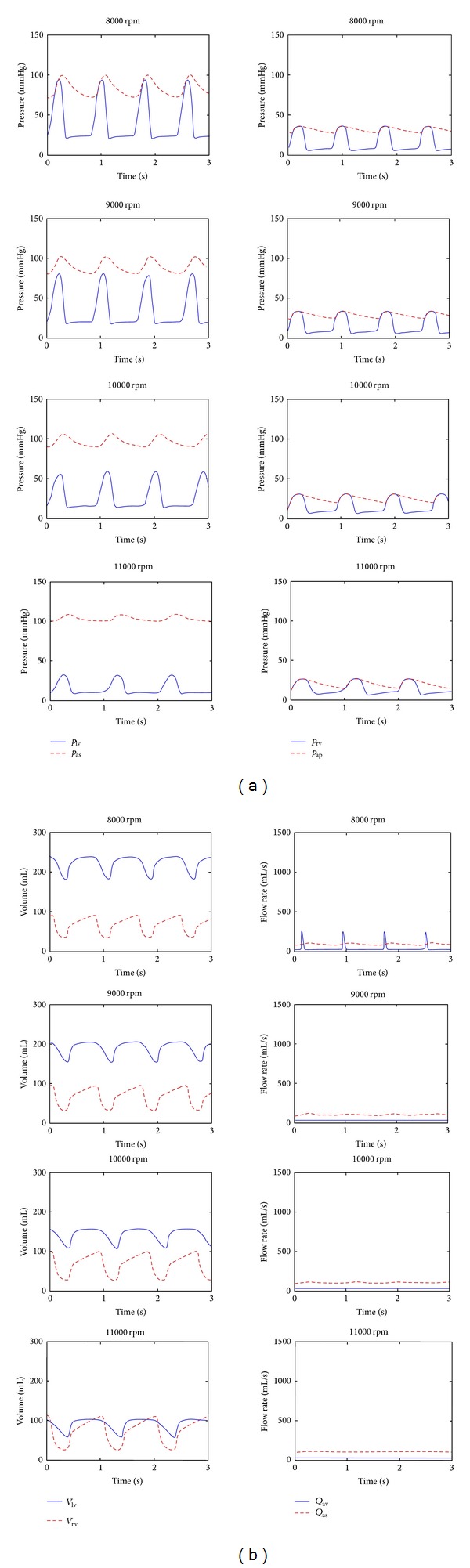
Pressures in the left ventricle (*p*_lv_), the systemic arteries (*p*_as_), the right ventricle (*p*_rv_), the pulmonary arteries (*p*_ap_) and volumes in the left ventricle (*V*_lv_) and the right ventricle (*V*_rv_) and flows through the aortic valve (*Q*_av_) and the systemic arteries (*Q*_as_) under HTA support.

HTA was operated between 8000 rpm and 11000 rpm operating speeds with 1000 rpm intervals in the CF-LVAD integrated cardiovascular system model. The pressures in the ventricles and systemic and pulmonary arteries and ventricular volumes under HTA support are given in Figure 4.

Left ventricular peak pressure becomes lower at relatively higher pump operating speeds in a CF-LVAD assisted failing heart. Also it is expected to have lower end-diastolic left ventricular pressure under relatively high pump operating speeds due to lowered preload [28, 29]. HTA support caused a decrease in the end-systolic and end-diastolic left ventricular pressures. Under the CF-LVAD support, the end-systolic left ventricular pressure was 30 mmHg at 11000 rpm HTA operating speed. Further increase in the pump speed may cause collapse in the left ventricle due to suction of the ventricular wall [28–30].

The aim of supporting the impaired left ventricle with CF-LVAD is to increase the blood flow and mean arterial pressure to maintain the normal organ function in the body. There was an upward shift in the systemic arterial pressure signal that indicates the increased mean pressure in the systemic arteries under HTA support. However, amplitude of the systemic arterial pressure signal decreased under support of HTA. Left ventricular pressure remained below arterial pressure over a cardiac cycle at the pump speeds higher than 8000 rpm.

High peak pressures in the right ventricle and the pulmonary arteries reduced under HTA support. HTA support did not have a significant effect on the end-diastolic right ventricular pressure like the amplitude of pulmonary arterial pressure signal.

Left ventricular volume, which was higher than 200 mL in the DCM model, decreased significantly under HTA support. Left ventricular volume was in the healthy physiological range at 10000 rpm HTA operating speed [31]. The stroke volume decreased due to continuous unloading of the left ventricle over a cardiac cycle under HTA support. Relatively higher operating speeds caused more reduction in the left ventricular stroke volume. Right ventricular volume increased with respect to DCM model under HTA support. Right ventricular stroke volume also increased due to increased mean pump output at the relatively higher HTA speeds.

The heart and the HTA operated together at 8000 rpm operating speed. The amplitude of the aortic valve flow signal was 250 mL/s at this speed. The aortic valve remained close over a cardiac cycle at the relatively higher operating speeds due to high pressure rise at the outlet of HTA. Therefore, there was no ejection through the aortic valve at these operating speeds. The pulsatility in the arterial flow was decreased due to lower left ventricular pressure with increasing pump speeds.

**Figure 5:**
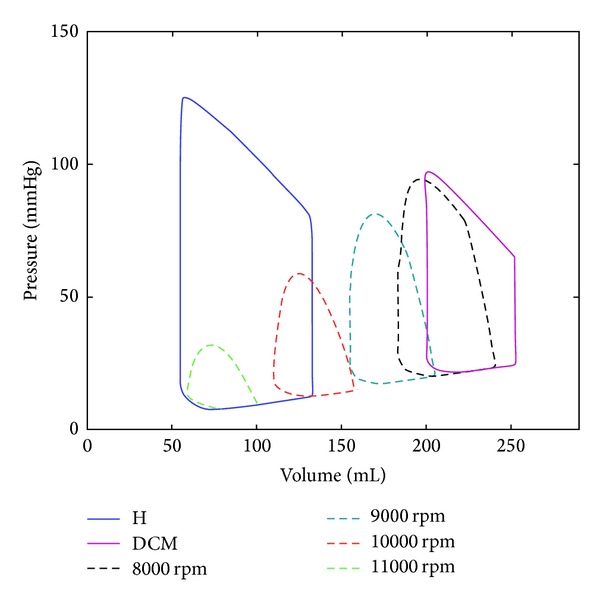
Left ventricular pressure-volume loops for the healthy (*H*) and dilated cardiomyopathic (DCM) and HTA supported models between 8000 rpm and 11000 rpm operating speeds with 1000 rpm increments.

Left ventricular pressure-volume loops give the information about the unloading of the left ventricle under CF-LVAD support. Left ventricular pressure volume loops under HTA support for the simulated operating speeds are given in Figure 5.

Left ventricular pressure volume loop shifted to right because of the increased cavity volume in the DCM model. There was a reduction in its area because of the reduced systolic left ventricular pressure and stroke volume. Left ventricular pressure volume loop area which indicates the left ventricular stroke work is expected to decrease under CF-LVAD support due to continuous unloading of this heart chamber by the pump. Left ventricular stroke work decreased with increasing HTA operating speed due to higher level of unloading at the relatively higher speeds. Isovolumic contraction and relaxation phases disappeared due to continuous unloading of the ventricle under CF-LVAD support. Pressure volume loop area of the left ventricle was very small at 11000 rpm operating speed. This also indicates that further increase in the HTA operating speed may cause collapse of the ventricle due to suction.

**Figure 6:**
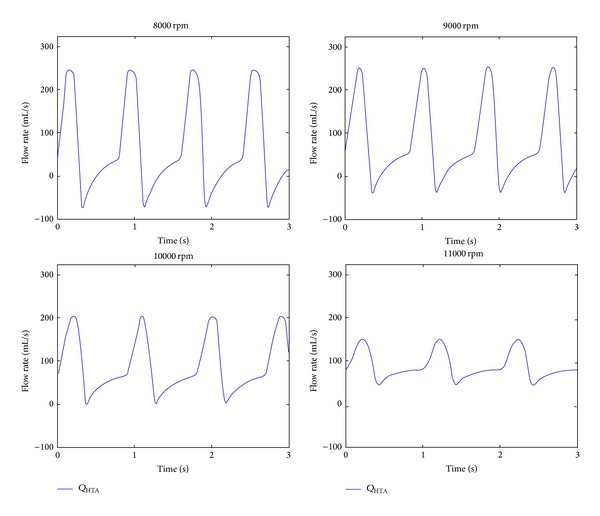
Flow through HTA (*Q*_HTA_) between 8000 rpm and 11000 rpm operating speeds with 1000 rpm increments.

Flow rate through the pump is an important parameter in a CF-LVAD supported heart. Blood flow should not regurgitate through the pump for an efficient CF-LVAD support. This happens at the relatively lower pump operating speeds. At the relatively higher speeds, flow rate signal becomes less pulsatile. In the excessive pumping, left ventricle may collapse and flow rate signal through the pump may be distorted over a cardiac cycle. Flow rate through HTA between 8000 rpm and 11000 rpm pump operating speeds with 1000 rpm is increments given in Figure 6.

Blood flow through HTA regurgitates at 8000 rpm and 9000 rpm operating speeds. At 10000 rpm regurgitation of the blood flow disappeared. However, the amplitude of the flow rate signal decreased. The amplitude of the flow rate signal was minimum at 11000 rpm HTA operating speed.

**Figure 7:**
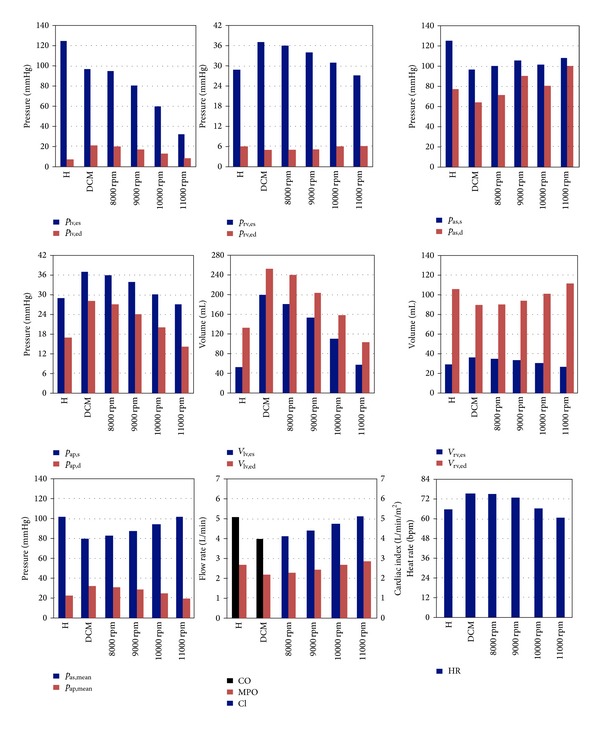
Comparison of the hemodynamic variables in the healthy (*H*), dilated cardiomyopathic (DCM), and HTA assisted models.  *V*  and  *p*  denote the volume and pressure respectively. CO, MPO and CI are the cardiac output, mean pump output, and cardiac index, and HR is the heart rate. Subscripts lv, rv, as, and ap represent the left ventricle, the right ventricle, the systemic arteries and the pulmonary arteries,  es  and ed denote end-systolic and end-diastolic, and  *s*  and  *d*  are systolic and diastolic, respectively.

The hemodynamic variables at the end of the systole and at the end of the diastole, together with the heart rate, mean pump output and the cardiac index for the healthy, DCM, and HTA supported cardiovascular system models is given in Figure 7.

End-systolic pressure in the left ventricle decreased due to impaired contraction. Left ventricular end-systolic pressure decreased more under assistance of HTA due to continuous unloading of the left ventricle. High end-diastolic volume in the DCM model decreased under HTA support. It was 8 mmHg, at 11000 rpm HTA operating speed. End-systolic right ventricular pressure increased because of lowered afterload in the DCM model. This hemodynamic variable was reduced to below healthy level at 11000 rpm operating speed. In the DCM model, decrease of the end-diastolic right ventricular pressure was low with respect to healthy model. This parameter reached the healthy level at 10000 rpm operating speed under HTA support.

At 8000 rpm pump operating speed, the mean arterial pressure became 83 mmHg by increasing 3 mmHg with respect to the DCM model. Under relatively higher speed CF-LVAD support, the systolic and mean arterial pressures increased more. However, the amplitude of the arterial pressure signal reduced which indicates a decrease in the arterial pressure pulsatility. The mean arterial pressure was 103 mmHg which was the same as the healthy level at 11000 rpm HTA operating speed. Mean pump output was at the same level as the healthy model at 11000 rpm HTA operating speed as well as the cardiac index. Keeping the mean arterial pressure and ejected mean blood at a certain level is very important to maintain the proper organ functions. Therefore, these parameters should be at the healthy level in mechanical circulatory device supported cardiovascular system. This parameter increased to level in the healthy model at 11000 rpm HTA operating speed.

High systolic pulmonary arterial pressure in the DCM model reduced to healthy level at 10000 rpm HTA operating speed. Pulmonary arterial pressure at the diastole in DCM model was higher than the healthy model. This parameter decreased to healthy level at 10000 rpm HTA operating speed. The high end-systolic and end-diastolic volume in the left ventricle decreased under HTA support. The end-systolic volume reduced to the healthy level at 11000 rpm HTA speed while end-diastolic volume was lower than the level in healthy model. The end-diastolic volume increased while the end-systolic volume decreased with respect to DCM model under HTA support.

In a DCM patient heart rate increases for maintaining the cardiac output and systemic arterial as in the healthy situation. In the DCM model heart rate became 75 bpm increasing by 10 bpm with respect to healthy model. HTA support caused a decrease in this parameter by increasing the afterload. At 10000 rpm operating speed, heart rate was 67 bpm.

In this study, a numerical model was developed for describing the pressure-flow rate relation of a new CF-LVAD to evaluate the hemodynamic response of a DCM cardiovascular system model under support of this CF-LVAD. The CFD analyses were performed for the operating speeds between 5000 rpm and 15000 rpm. The reverse flow rate-pressure relation in the pump was assumed to have the same relation as the forward flow rate-pressure relation.

The results also give an insight about the possible operating speed interval of this CF-LVAD in a patient. At 11000 rpm HTA operating speed the mean arterial pressure and, the mean pump output reached the same level as in the healthy model. However, left ventricular peak pressure was very low at this speed. The blood flow through HTA regurgitated at the operating speeds below 10000 rpm because of the relatively high pressure at the outlet of the pump. This should be avoided for achieving an efficient mechanical circulatory support in the patient. Therefore HTA should not operate at the speeds lower than 10000 rpm in a patient.

Existence of the baroreflex model allowed us to evaluate the response of the cardiovascular system model with respect to changing heart rate. The effect of the CF-LVAD on the hemodynamical parameters without the model of baroreflex would be slightly different because of keeping the heart rate constant. This model also showed the effect of the HTA support on the heart rate.

In a DCM patient, the total blood volume increases for maintaining the perfusion level [32]. We did not include that regulatory mechanism in the model. For introducing such a mechanism to describe the change of the blood volume, the renal function must be identified in the DCM and CF-LVAD supported patients. Identifying such a mechanism needs elaborated experiments and system identification tools. However, it should be noted that the mathematical model used for describing the cardiovascular system was able to produce healthy and DCM dynamics with a good agreement with the data presented in the literature [31].

In a mechanical circulatory assist device supported heart myocardium recovers due to support. However, this recovery is not sufficient to maintain the perfusion without CF-LVAD assistance [33]. In this study, myocardial recovery is not included in the HTA supported model due to the fact that the exact mechanism is still unknown [33].

## 4. Conclusions

Normally, there is a range for the optimum operating speed for such a device in the clinical applications and the optimal pump speed is determined according to the hemodynamical data monitoring the cardiac index and the left ventricle size in a patient [14]. The findings in the simulation study provide us useful guidelines in testing of this new CF-LVAD. The minimum operating speed should be 10000 rpm in a patient due to reverse flow through the pump at the relatively lower operating speeds. Based on the findings, the range of the HTA operating speed should be between 10000 rpm and 11000 rpm in order to achieve the perfusion for maintaining organ functions in the clinical applications.

## Figures and Tables

**Table 1:** Parameters used in the blood vessel and ventricle models [18–21].

	*R* (mmHg·s/mL)	*L* (mmHg·s^2^/mL)	*C* (mL/mmHg)	*E*_*s*_ (mmHg/mL)
Left ventricle	—	—	—	2.5
Left atrium	—	—	5	
Mitral valve	5e − 3	—	—	
Aortic valve	8e − 3	—	—	
Systemic arteries	1	1e − 5	1.5	
Systemic veins	0.09	1e − 5	82.5	
Right ventricle	—	—	—	1.15
Right atrium	—	—	5	
Tricuspid valve	5e − 3	—	—	
Pulmonary valve	3e − 3	—	—	
Pulmonary arteries	9e − 2	1e − 5	6.5	
Pulmonary veins	7e − 2	1e − 5	5	
