# Corrigendum to “Evaluating the Hemodynamical Response of a Cardiovascular System under Support of a Continuous Flow Left Ventricular Assist Device via Numerical Modeling and Simulations”

**DOI:** 10.1155/2019/2906543

**Published:** 2019-09-03

**Authors:** Selim Bozkurt, Koray K. Safak

**Affiliations:** Department of Mechanical Engineering, Yeditepe University, Kadikoy, Istanbul 34755, Turkey

In the article titled “Evaluating the Hemodynamical Response of a Cardiovascular System under Support of a Continuous Flow Left Ventricular Assist Device via Numerical Modeling and Simulations” [1], there were errors in equations (4) and (7) that should be corrected as follows:

1. In equation (4), the left ventricular volume change (*dV*_lv_*/dt*) was described as the difference between flow rates through the mitral valve (*Q*_mv_) and aortic valve (*Q*_av_). The equation should be as follows:

(4) $$\begin{array}{c} \frac{dV_{\text{lv}}}{dt}=Q_{\text{mv}}-Q_{\text{av}}. \end{array}$$

(2) In equation (7), the HTA model and the circulatory system model were combined to simulate the CF-LVAD assistance using the equations which describe the left ventricular volume change (*dV*_lv_*/dt*) and systemic arterial pressure change (*dp*_as_/*dt*). The equation should be corrected as follows:

(7) $$\begin{array}{c} \frac{dV_{\text{lv}}}{dt}=Q_{\text{mv}}-Q_{\text{av}}-Q_{\text{HTA}},\\ \frac{dp_{\text{as}}}{dt}=\frac{Q_{\text{av}}+Q_{\text{HTA}}-Q_{\text{as}}}{C_{\text{as}}}. \end{array}$$

These equations were used correctly in the computations and errors appear only in the text.
